# Supplementary material for: G2PDeep-v2: A Web-Based Deep-Learning Framework for Phenotype Prediction and Biomarker Discovery for All Organisms Using Multi-Omics Data
Source: Biomolecules. 2025 Dec 1;15(12):1673. doi: 10.3390/biom15121673 (PMC12730281; doi:10.3390/biom15121673)
Supplement: Supplementary file 1 [file biomolecules-15-01673-s001.zip › supplementary Fig. S1.pdf]

Supplementary information for

G2PDeep-v2: a web-based deep-learning framework for phenotype prediction and biomarker discovery using multi-omics data

Shuai Zeng[1,2], Trinath Adusumilli[1], Sania Zafar Awan[3], Manish Sridhar Immadi[1], Dong Xu[1,2,3] and Trupti Joshi[1,2,3,4,*]
[1] Department of Electrical Engineering and Computer Science, University of Missouri, Columbia, MO, 65211, USA
[2] Christopher S. Bond Life Sciences Center, University of Missouri, Columbia, MO, 65211, USA
[3] MU Institute for Data Science and Informatics, University of Missouri-Columbia, Columbia, MO, 65211, USA
[4] Department of Biomedical Informatics, Biostatistics and Medical Epidemiology, University of Missouri, Columbia, MO, 65211, USA
* To whom correspondence should be addressed. Tel: +1 (573) 884-5963; Email: joshitr@health.missouri.edu

Email:
Shuai Zeng, zengs@missouri.edu
Trinath Adusumilli, tafbr@missouri.edu
Sania Zafar Awan, sah2p@missouri.edu
Manish Sridhar Immadi, mizy9@missouri.edu
Dong Xu, xudong@missouri.edu
Trupti Joshi, joshitr@health.missouri.edu

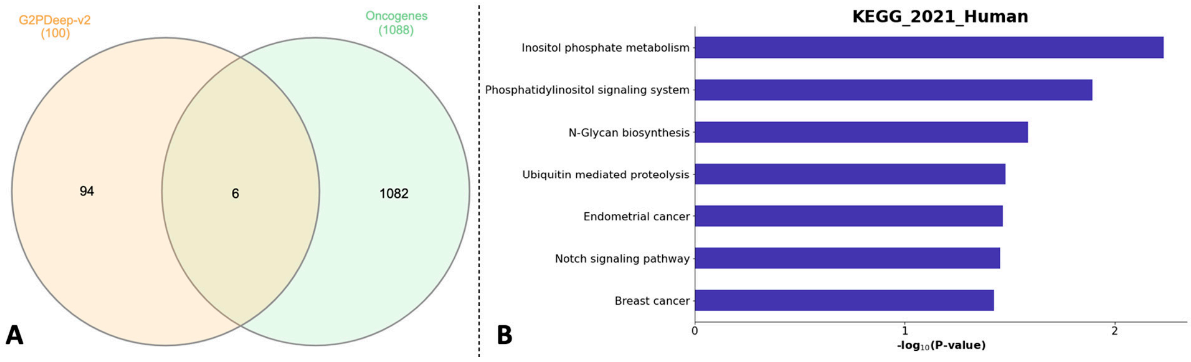

Supplementary Fig. 1. Plots for significant biomarkers. (A) Venn diagram of 100 most significant biomarkers and oncogenes. (B) Enriched seven pathways associated with breast cancer.
